# The Relationship Between Preoperative Serum Ionized Calcium, Vitamin D, and Postoperative Bleeding After Major Cardiac Surgery

**DOI:** 10.3390/life15091460

**Published:** 2025-09-17

**Authors:** Adrian Stef, Constantin Bodolea, Aurelia Georgeta Solomonean, Nadina Tintiuc, Alexandru Oprea, Oana Antal, Gabriel Cismaru, Emanuel Palade

**Affiliations:** 1 Clinical Department of Anesthesia and Intensive Care, Heart Institute “Niculae Stancioiu”, “Iuliu Hatieganu” University of Medicine and Pharmacy, Motilor 19-21, 400001 Cluj-Napoca, Romania; stef.adrian@yahoo.com (A.S.); ntintiuc@yahoo.com (N.T.); 2 Anesthesia and Intensive Care 2 Discipline, “Iuliu Hatieganu” University of Medicine and Pharmacy, Victor Babes Nr 8 Street, 400012 Cluj-Napoca, Romania; constantin.bodolea@umfcluj.ro (C.B.); contact@umfcluj.ro (O.A.); 3 Cardiothoracic Surgery Department, “Iuliu Hatieganu” University of Medicine and Pharmacy, 400337 Cluj-Napoca, Romania; alexandru_oprea2002@yahoo.com; 4 Department of Cardiovascular Surgery, Heart Institute “Niculae Stancioiu”, 400001 Cluj-Napoca, Romania; 5 4th Department of Internal Medicine, Cardiology Rehabilitation, “Iuliu Hatieganu” University of Medicine and Pharmacy, Victor Babes Nr.8 Street, 400012 Cluj-Napoca, Romania; 6 Thoracic Surgery Clinic, Leon Daniello Clinical Hospital of Pneumology, 400371 Cluj-Napoca, Romania; 7 Department of Surgery, “Iuliu Hatieganu” University of Medicine and Pharmacy, 400000 Cluj-Napoca, Romania

**Keywords:** ionized calcium, cardiopulmonary bypass, major cardiac surgery, bleeding

## Abstract

**Objective:** The role of calcium in coagulation homeostasis is well established, although the relationship between calcium levels and postoperative bleeding in major cardiac surgery remains largely unexplored. **Methods:** This retrospective, single-center study investigated the correlations between ionized calcium levels measured at several timepoints: preoperatively (after induction of anesthesia), immediately after cardiopulmonary bypass (CPB) weaning, on the first postoperative day measured three times at 8 h intervals, preoperative vitamin D values, and several significant bleeding outcomes. These outcomes included the volume of blood in the drainage bag (measured in milliliters on days 1 and 2), the need for surgical or medical hemostasis, the requirement for blood transfusion (red blood cells, fresh frozen plasma, or platelets), and the occurrence of extracardiac hemorrhagic complications. A multivariable logistic regression analysis was performed, with a two-sided *p*-value of <0.00625 considered significant after applying Bonferroni correction. **Results:** The study included 83 patients with a mean age of 64.9 ± 8.5 years, with 49 (59%) being male. The most common procedures were aortic valve replacement (26 patients, 31%) and coronary artery bypass grafting (26 patients, 31%). The multivariable regression analysis demonstrated a trend toward an association between low levels of preoperative calcium and increased bleeding volume immediately after CBP and on the first day after the intervention (r = 0.30; *p* = 0.08 for day 1 and r = 0.24; *p* = 0.03 for day 2). Similar trends were observed for the association between low levels of preoperative calcium, use of medical hemostasis (r = 0.30; *p* = 0.009), and red blood cell transfusion (r = 0.24; *p* = 0.03). Additionally, we observed a trend towards a positive correlation between lower serum vitamin D levels and increased postoperative blood loss on both day 1 (r = 0.32; *p* = 0.07) and day 2 (r = 0.29; *p* = 0.04). The subgroup analysis of valve procedures vs. coronary procedures showed no statistically difference between preoperative ionized calcium levels, postoperative bleeding (289 27 vs. 283 mL, *p* = 0.87), the need for surgical hemostasis (*p* = 0.5), or blood transfusion requirement (*p* = 0.57). **Conclusions:** In our study, preoperative calcium levels were consistently associated with increased bleeding after major cardiac surgery. Post-CPB ionized calcium levels did not influence bleeding outcomes. The role of calcium in coagulation homeostasis during major cardiac surgery warrants further research, ideally with more robust data, as our study’s small sample limits robust evidence. Further larger studies will conclude on the importance of calcium levels in cardiac surgery related to hemostasis and bleeding outcomes. Lower preoperative ionized calcium and vitamin D levels showed exploratory associations with increased bleeding-related outcomes following major cardiac surgery. These findings are hypothesis-generating, and larger prospective studies are needed to confirm these potential relationships and clarify their clinical implications.

## 1. Introduction

Calcium is a pivotal element in the coagulation cascade, functioning as a cofactor in numerous enzymatic reactions that are essential for blood clot formation and stability. In major cardiac surgery, particularly those involving cardiopulmonary bypass (CPB), calcium homeostasis can be significantly disrupted due to hemodilution, alterations in pH, and the use of citrate-containing anticoagulants [1,2,3]. These disruptions have led to an ongoing interest in understanding how fluctuations in ionized calcium levels during and after CPB might influence surgical outcomes, particularly in relation to bleeding complications [4,5].

The role of calcium in major cardiac surgery extends beyond its involvement in coagulation. Hypocalcemia, which commonly occurs during CPB, has been associated with hemodynamic instability, reduced myocardial contractility, and arrhythmias, all of which can adversely affect surgical outcomes. Conversely, hypercalcemia, although less common, may predispose patients to hypercoagulability, increasing the risk of thrombotic events. Given these potential consequences, the management of calcium levels during and after CPB is critical, yet the optimal approach remains a subject of debate [6].

Recent studies have aimed to elucidate the impact of calcium management on clinical outcomes in cardiac surgery. Li et al. (2024) investigated calcium levels during heart valve surgery and their effects on coagulation and bleeding, finding that hypocalcemia affected bleeding outcomes [7]. Similarly, Vasudeva et al. (2021) reported moderate-quality evidence on the association between transfusion-independent hypocalcemia and mortality, blood transfusion needs, and coagulopathy [8].

Despite these findings, the specific relationship between ionized calcium levels and clinical bleeding outcomes remains inadequately explored, particularly in the early postoperative period, when patients are in the intensive care unit (ICU). Bleeding remains a significant complication following major cardiac surgery, contributing to increased morbidity, mortality, and healthcare costs. Therefore, it is crucial to identify modifiable factors, such as calcium levels, that could potentially mitigate bleeding risks.

The objective of this research was to investigate whether ionized calcium levels correlate with specific bleeding outcomes and to assess correlations between preoperative vitamin D levels and bleeding outcomes after major cardiac surgery requiring CPB.

Red blood cell (RBC) transfusion is a cornerstone in the management of patients undergoing cardiac surgery, particularly in those experiencing significant blood loss. The strategy employed—whether restrictive or liberal—can significantly influence patient outcomes. Traditionally, transfusion decisions have been guided by hemoglobin (Hb) thresholds. However, recent studies suggest that a more physiological approach, considering markers of organ hypoperfusion, may be beneficial. For instance, a systematic review and meta-analysis by Putaggio et al. indicated that restrictive RBC transfusion strategies are as safe as liberal strategies in patients undergoing cardiac surgery, with no significant difference in short-term clinical outcomes [9,10,11]. This approach not only aligns with current evidence but also emphasizes the importance of individualized patient care in transfusion practices.

## 2. Methods

### 2.1. Source of Data, Patient Population, Bleeding Parameters

This retrospective, single-center study investigated the correlation between ionized calcium levels, vitamin D levels, and various bleeding outcomes. Ionized calcium level was measured preoperatively (after induction of anesthesia), immediately after cardiopulmonary bypass (CPB) weaning, and on the first postoperative day three times at 8 h intervals. The vitamin D level was measured 2 h before surgery. Bleeding outcomes included the volume of blood in the drainage bag on days 1 and day 2 after surgery, the need for surgical or medical hemostasis, the requirement for blood transfusion (red blood cells, fresh frozen plasma, or platelets), and the occurrence of extracardiac hemorrhagic complications. A multivariable logistic regression analysis was performed. To account for multiple comparisons, Bonferroni correction was applied, adjusting the threshold for statistical significance to a two-sided *p*-value of <0.00625 (i.e., 0.05 divided by 8 tests).

As an exploratory analysis, we derived a receiver operating characteristic (ROC) curve to evaluate the potential preoperative ionized calcium threshold associated with increased risk of postoperative bleeding. Excessive bleeding was defined as drainage volume above the 75th percentile of our cohort or requirement for red blood cell transfusion. The area under the curve (AUC) was calculated, and the optimal cutoff value was determined using the Youden index.

Inclusion criteria were patients over 18 years old admitted to the Niculae Stăncioiu Heart Institute in Cluj-Napoca who underwent elective cardiovascular procedures requiring general anesthesia and cardiopulmonary bypass during 1 October 2021 to 28 February 2022. The current study was approved by the Iuliu Haţieganu University of Medicine Ethics Committee (approval 259 on 28 September 2023). Our research was conducted in accordance with the principles of the Declaration of Helsinki and good clinical practice guidelines. Considering the retrospective nature of the study and minimal risk to participants, there was no need for individual patient consent.

The patients were enrolled in a single database, where demographic and clinical characteristics were recorded from our internal hospital database. Data were analyzed retrospectively and checked against the hospital information system.

Exclusion criteria included urgent indications, patients with hemodynamic instability referred for major cardiovascular surgery, individuals with a history of hyperparathyroidism or active neoplasia, calcium or vitamin D supplement intake prior to hospitalization, inadequate blood collection protocols, inadequate drainage recording, and patients with a hospital length of stay less than 24 h (including those who were transferred to other facilities).

Ionized calcium levels were assessed preoperatively (after induction of anesthesia), immediately after CBP weaning, and every 8 h within the first 24 h after surgery. The values were indirectly calculated from the value of serum calcium and total protein (TP) according to the following formula: Ca++ = (6 × Ca − TP/3):(TP + 6)^3^. This method has been reported in prior studies as a surrogate when direct ionized calcium assays are unavailable, although it is acknowledged to be less precise, particularly in patients with altered protein levels.

In our laboratory, the reference ranges for ionized calcium are 1.05–1.3 mmol/L, and normal vitamin D levels are defined as ranging from 30 to 50 ng/mL. Vitamin D deficiency is defined by values ranging from 20 to 30 ng/mL. Deficiency in vitamin D is defined by values below 20 ng/mL. Vitamin D levels were stratified as deficient (<20 ng/mL), insufficient (20–30 ng/mL), and normal (30–50 ng/mL) based on standard clinical thresholds. Stratified analyses were performed to explore potential differences in bleeding outcomes across these categories.

The levels of vitamin D were assessed 2 h prior to the surgical procedure. All measurements were conducted using identical instruments, specifically the Biotek Microplate 50 TS washer and the 800 TS reader, both from Agilent Technologies Inc. (Santa Clara, CA, USA).

The bleeding parameters were assessed and recorded by two independent ICU physicians. Blood in the mediastinal drainage tube was measured in milliliters as per the drainage bag labeling every 24 h. The need for surgical hemostasis was defined as surgical reintervention or any surgical maneuver performed by the attending cardiac surgeon with the aim of stopping a bleeding complication. The need for medical hemostasis was defined as the administration of hemostatic drugs: antifibrinolytics, prothrombin complex concentrate, activated X factor, fibrinogen, or protamine. All patients received tranexamic acid as a bolus of 15 mg/kg in the preoperative and in the postoperative periods. Extracardiac hemorrhagic complications were defined as active bleeding from the surrounding tissues and organs not related to the cardiac procedure itself (pulmonary, abdominal, etc.).

### 2.2. Statistical Methods

Continuous variables are described using means ± standard deviation, while categorical variables are described using counts and proportions. Relationships between the exposures of interest and binary outcomes were modeled using multivariable logistic regression, with odds ratios (ORs) and 95% confidence intervals (CIs) the summary statistics. Relationships between the exposures of interest and continuous outcomes were modeled using multivariable linear regression, with coefficients (β) and 95% CIs the summary statistics. All multivariable regressions were adjusted for age, body mass index, sex, type of procedure, and duration of cardiopulmonary bypass, all of which were prespecified based on clinical knowledge and potential causal pathways.

To assess whether preoperative ionized calcium is an independent predictor of postoperative bleeding, we performed multivariable regression analyses. The dependent variables included clinically relevant bleeding outcomes: total drainage volume on postoperative days 1 and 2, requirement for red blood cell transfusion, and need for medical hemostasis. The independent variables were preoperative ionized calcium levels, age, sex, body mass index, type of procedure (valve, coronary, or combined), and duration of cardiopulmonary bypass. These covariates were chosen based on clinical relevance and potential influence on postoperative bleeding.

Regression results were interpreted using Bonferroni-corrected significance thresholds (*p* < 0.00625) to account for multiple comparisons. This approach allows evaluation of whether preoperative calcium is an independent predictor of bleeding outcomes while controlling for important confounders.

A post hoc power analysis was conducted for the primary continuous outcome (postoperative drainage volume). With a sample size of 83 patients, an alpha level of 0.05, and a standard deviation of approximately 200 mL (based on the observed data), the study had 80% power to detect a mean difference of ~90 mL in drainage volume between groups. Smaller effect sizes would not have been reliably detected.

A post hoc power analysis was performed. For the continuous outcome of postoperative drainage volume, using the observed standard deviation (~200 mL), a total sample of 83 patients, and α = 0.05, the study had approximately 80% power to detect a between-group mean difference of ~90 mL. For the binary outcome of red blood cell transfusion (observed event rate 33.7%), assuming a two-group comparison with roughly equal group sizes (e.g., low vs. normal preoperative ionized calcium) and a two-sided test, the study achieved ~80% power only for large differences (≈34% vs. ≈66%; odds ratio ~3.8) or substantial decreases (≈34% vs. ≈10%; odds ratio ~0.22) at α = 0.05. When applying the Bonferroni-adjusted threshold (α = 0.00625), the detectable differences became even larger (≈34% vs. ≈72%; odds ratio ~6.2, or ≈34% vs. ≈5%; odds ratio ~0.10).

In multivariable analyses, lower preoperative ionized calcium levels showed a non-significant trend toward increased postoperative drainage volume, higher likelihood of red blood cell transfusion, and greater need for medical hemostasis. For example, the association between preoperative calcium and day 1 drainage volume had a nominal *p*-value of 0.03 and the association with medical hemostasis had a nominal *p*-value of 0.009; however, neither reached the Bonferroni-corrected significance threshold of *p* < 0.00625.

These findings suggest that preoperative calcium may act as an independent predictor of postoperative bleeding, though this conclusion is hypothesis-generating due to limited sample size and the observational nature of the study. Other clinically relevant factors, such as preoperative anemia, platelet count, or surgical complexity, may also contribute to bleeding outcomes and should be incorporated in future larger prospective studies.

Hypothesis testing performed for each exposure variable was considered to be separate sets of tests in terms of considerations for multiple testing. To mitigate inflation of type I error due to multiple testing, Bonferroni correction was used, with two-sided *p* < 0.00625 deemed statistically significant. All analyses were performed using Stata 16.1 (StataCorp LLC, College Station, TX, USA).

## 3. Results

The study included 83 patients who underwent major cardiac surgery. The mean age of the patients was 64.9 ± 8.5 years, and 49 patients (59%) were male. The most common procedures were aortic valve replacement (26 patients, 31%) and coronary artery bypass grafting (26 patients, 31%). An exploratory association was observed between lower preoperative ionized calcium levels and bleeding volume immediately after CBP (r = 0.30; *p* = 0.08) and in the first 24 h after surgery (r = 0.24; *p* = 0.03), although this did not meet the Bonferroni-adjusted threshold for statistical significance.

A trend towards an association was found between low levels of preoperative ionized calcium, use of medical hemostasis (r = 0.30; *p* = 0.009), and use of red blood cell transfusion (r = 0.24; *p* = 0.03). Additionally, a similar trend was found between lower serum vitamin D levels and increased postoperative blood loss immediately after CBP (r = 0.32; *p* = 0.07) and in the first 24 h after surgery (r = 0.29; *p* = 0.04).

Altogether, 83 patients were analyzed after applying the inclusion and exclusion criteria. Their baseline characteristics are summarized in Table 1.

The volume of drainage on postoperative day 1 was 425 ± 215 mL, and that on postoperative day 2 was 294 ± 167 mL. In total, 4 patients required surgical hemostasis (4.8%), 28 required red blood cell transfusion (33.7%), 21 required fresh frozen plasma transfusion (25.3%), 8 required platelet transfusion (9.6%), and 14 required medical hemostasis (16.9%).

A non-significant trend was observed between lower preoperative ionized calcium levels (1.16–1.31 mmol/L) and increased bleeding volume immediately after CPB (r = 0.30; *p* = 0.08) and on the first postoperative day (r = 0.24; *p* = 0.03). Similarly, there was a suggestive association between lower preoperative calcium levels and the need for medical hemostasis (r = 0.30; *p* = 0.009) and red blood cell transfusion (r = 0.24; *p* = 0.03). However, none of these associations reached the Bonferroni-corrected significance threshold of *p* < 0.00625.

Lower serum vitamin D levels showed a similar non-significant trend with increased postoperative blood loss immediately after CPB (r = 0.32; *p* = 0.07) and on the first postoperative day (r = 0.29; *p* = 0.04), as well as with increased need for red blood cell transfusion (r = 0.23; *p* = 0.04) and medical hemostasis (r = 0.27; *p* = 0.02). Again, these results did not meet the adjusted significance threshold.

Postoperative ionized calcium levels measured immediately after CPB (mean 1.165 mmol/L; 95% CI 1.151–1.180) and during the first 24 h postoperatively (mean 1.197 mmol/L; 95% CI 1.185–1.210) showed no statistically significant associations with any bleeding outcome (all *p* > 0.00625).

Subgroup analysis of valve versus coronary procedures revealed no significant differences in preoperative ionized calcium or vitamin D levels, postoperative bleeding, surgical or medical hemostasis requirements, or transfusion needs. Trends linking lower preoperative calcium to increased bleeding and transfusion requirements were observed in both subgroups, but did not reach Bonferroni-corrected significance.

Exploratory ROC analysis suggested that a preoperative ionized calcium level of approximately 1.15 mmol/L may be associated with increased risk of excessive postoperative bleeding (AUC = 0.65, 95% CI 0.53–0.77). While this threshold is hypothesis-generating and does not reach statistical significance after Bonferroni correction, it highlights potential value for identifying patients at higher bleeding risk.

Exploratory associations were observed between lower preoperative ionized calcium levels and bleeding volume immediately after CPB (r = 0.30; *p* = 0.08) and within the first 24 h after surgery (r = 0.24; *p* = 0.03). Similar exploratory associations were also noted with the use of medical hemostasis (r = 0.30; *p* = 0.009) and red blood cell transfusion (r = 0.24; *p* = 0.03). Lower serum vitamin D levels also showed exploratory associations with increased postoperative blood loss immediately after CPB (r = 0.32; *p* = 0.07) and during the first 24 h after surgery (r = 0.29; *p* = 0.04). These associations did not meet the Bonferroni-adjusted threshold for statistical significance and should therefore be interpreted as hypothesis-generating.

**Figure 1:**
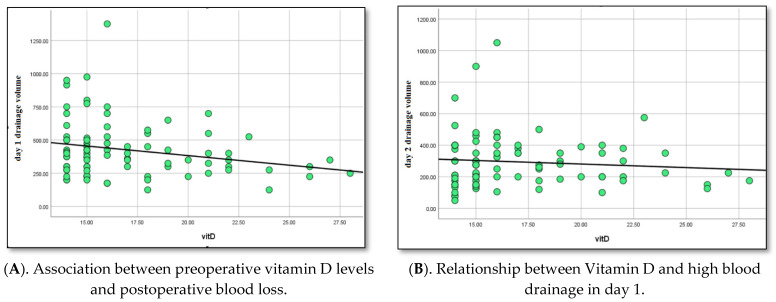
(**A**). Relationship between serum vitamin D levels and drainage volume immediately after cardiopulmonary bypass (CPB). Lower vitamin D levels showed a non-significant trend toward higher blood loss, but the association did not reach the Bonferroni-corrected significance threshold (*p* < 0.00625). (**B**). Relationship between serum vitamin D levels and drainage volume on the first postoperative day. Similarly, lower vitamin D levels demonstrated a suggestive trend toward increased bleeding, without meeting the corrected significance threshold. Interpretation: These trends suggest a potential relationship between vitamin D status and early postoperative bleeding, warranting further investigation in larger studies.

The data suggest a possible correlation between initial serum calcium levels and red blood cell transfusion requirement (r = 0.24; *p* = 0.03), and the need for medical hemostasis using antifibrinolytics or protamine (r = 0.30; *p* = 0.009). Furthermore, we found a positive correlation between lower serum vitamin D levels and increased postoperative blood loss immediately after CBP (r = 0.32; *p* = 0.07), (Figure 1A) and at day 1 (r = 0.29; *p* = 0.04) (Figure 1B), and between requirement for red blood cell mass (r = 0.23; *p* = 0.04) and medical hemostasis (r = 0.27; *p* = 0.02).

Upon subgroup analysis, it was found that patients who underwent valve procedures had similar serum vitamin D levels (17.0 vs. 17.6 ng/mL, *p* = 0.81) to those who underwent coronary procedures.

We found low values for vitamin D level in both groups and no significant differences in preoperative calcium level between the two groups. There were no statistically significant differences in serum calcium levels (1.22 vs. 1.22 ng/mL, *p* = 0.92) between the two groups.

In exploratory stratified analyses, patients with vitamin D deficiency (<20 ng/mL) had higher median postoperative drainage volumes and increased rates of RBC transfusion and medical hemostasis compared to those with insufficient or normal vitamin D levels. Additionally, we performed a stratified analysis according to vitamin D status (deficient <20 ng/mL, insufficient 20–30 ng/mL, and normal 30–50 ng/mL). This exploratory analysis indicated that patients with vitamin D deficiency (<20 ng/mL) had numerically higher postoperative drainage volumes and a greater need for red blood cell transfusions and medical hemostasis compared to those with insufficient or normal levels. These results are provided in a new supplementary table (Table S1). However, given the small sample in each subgroup, these findings should be interpreted cautiously and considered hypothesis-generating.

**Figure 2:**
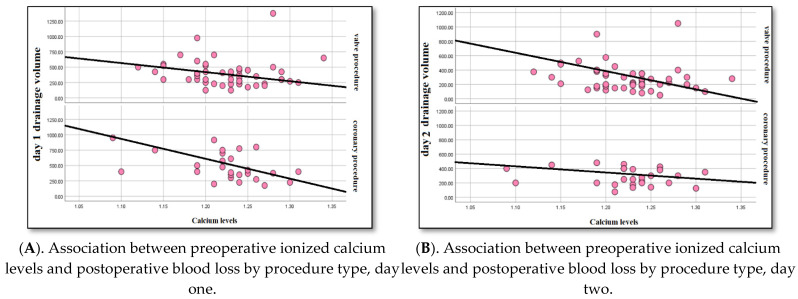
(**A**). Correlation between preoperative ionized calcium levels and drainage volume on the first postoperative day, stratified by procedure type (valve vs. coronary procedures). Lower calcium levels showed a non-significant trend toward higher blood loss in both subgroups. (**B**). Correlation between preoperative ionized calcium levels and drainage volume on the second postoperative day, stratified by procedure type. A similar non-significant trend was observed, with no associations reaching the Bonferroni-corrected significance threshold. Interpretation: These results suggest that lower preoperative calcium may be associated with increased bleeding in both valve and coronary procedures, although the associations were not statistically significant after correction for multiple comparisons.

There were no differences in postoperative bleeding (289 vs. 283/mL, *p* = 0.87), surgical and medical hemostasis (*p* = 0.5; *p* = 0.14), or transfusion need (*p* = 0.57) between the subgroups. A low preoperative ionized calcium level was linked to more frequent bleeding in both groups: valve group procedure (r = 0.33; *p* = 0.06) and coronary group procedure (r = 0.34; *p* = 0.05) (Figure 2A,B). In the valve group procedure, a low preoperative ionized calcium level was linked to a high blood cell transfusion need (r = 0.25; *p* = 0.004) and more medical hemostasis (r = 0.27; *p* = 0.015). Also, in the coronary group procedure, low preoperative ionized calcium level was linked to an increased need for red blood cell transfusion (r = 0.23; *p* = 0.04) and medical hemostasis (r = 0.28; *p* = 0.017).

Results from multivariable regressions are summarized in Table 2.

There were no statistically significant associations between the outcomes and postoperative ionized calcium levels (measured after CPB or on the first postoperative day) when applying the Bonferroni-corrected threshold (*p* < 0.00625). While some associations between postoperative ionized calcium and drainage blood volume on postoperative days 1 and 2 showed *p*-values below 0.05 (e.g., *p* = 0.011 and *p* = 0.016), these did not meet the stricter adjusted significance level and were therefore interpreted as nonsignificant.

Results from multivariable linear and logistic regression analyses are presented for postoperative ionized calcium levels measured immediately after cardiopulmonary bypass (CPB) and during the first postoperative day. All models were adjusted for age, body mass index, sex, type of procedure, and duration of CPB.

**Drainage volume (Day 1 and Day 2):** Negative coefficients indicated a potential inverse relationship between calcium levels and blood loss. Nominal *p*-values <0.05 are shown, but none reached the Bonferroni-corrected significance threshold of *p* < 0.00625, and should therefore be interpreted as non-significant trends.

**Surgical and medical hemostasis, transfusion requirements, and extracardiac hemorrhagic complications:** Odds ratios and coefficients indicated the estimated effect of postoperative calcium on these outcomes. None of the associations met the Bonferroni-corrected significance threshold, indicating no statistically significant relationships were observed.

**Interpretation:** Although some nominal associations suggest that lower postoperative calcium levels could be linked to higher bleeding or transfusion needs, these findings did not achieve statistical significance after correction for multiple comparisons. These results highlight potential trends that warrant further investigation in larger prospective studies.

## 4. Discussion

The primary finding of our study was that there were exploratory associations between preoperative ionized calcium levels and postoperative bleeding outcomes. Lower calcium levels were associated with greater bleeding within the first 48 h, including increased use of blood transfusion, and medical hemostasis, although these findings did not meet Bonferroni-adjusted significance thresholds. Similarly, lower vitamin D levels demonstrated exploratory associations with increased bleeding and greater need for hemostatic intervention during the early postoperative period. These findings should be interpreted cautiously and regarded as hypothesis-generating.

Although the ROC-derived threshold for preoperative ionized calcium is exploratory, it suggests a potential clinical cutoff that could help identify patients at higher risk of postoperative bleeding. This finding requires validation in larger prospective studies before it can be applied in clinical practice. Importantly, it complements our multivariable regression analyses by providing a practical perspective on calcium levels rather than replacing the adjusted models.

While calcium is known to play a crucial role in the coagulation cascade, serving as a cofactor in multiple steps of the process that leads to clot formation, vitamin D facilitates the active absorption of calcium from the small intestine. Inadequate calcium intake exacerbates the effects of vitamin D deficiency, and various levels of calcium intake demonstrate a correlation with the half-life of serum 25(OH)D levels [12]. During cardiac surgery, there is a transient decrease in calcium levels when the patient is placed on CPB, with values even slightly below the lower reference limit [13]. However, these levels tend to self-regulate immediately afterward. This phenomenon may disrupt calcium homeostasis, potentially impacting hemostasis and influencing postoperative bleeding.

There are very limited data exploring the relationship between calcium levels and bleeding outcomes in the context of cardiovascular surgery. A recent study by Li et al. (2024) evaluated the impact of ionized calcium levels on bleeding and transfusion requirements in patients undergoing cardiac surgery with CPB [7]. The authors found that lower ionized calcium levels after surgery (therefore after CPB) were associated with an increased risk of postoperative bleeding trough the drainage tube. Moreover, their study found an impressive difference in major bleeding rate between its hypocalcemia and normocalcemic groups (65% vs. 18%, *p* = 0.001) [7,8,9,14].

Although albumin-corrected total calcium is commonly used in clinical practice, we focused on ionized calcium, because it represents the physiologically active fraction and is more relevant for coagulation. Ionized calcium measurements are particularly important in the setting of cardiopulmonary bypass, where rapid shifts in pH, protein levels, and citrate can alter calcium availability. This approach allows a more accurate assessment of calcium’s potential impact on postoperative bleeding.

Calcium in clinically manifest bleeding has been studied more deeply in other fields, such as hemorrhagic strokes. Inoue et al. (2013) published the first study to investigate the relationships between serum calcium levels at admission and clinical findings and outcomes of patients with acute intracerebral hemorrhage [15]. Their major new finding was that patients with low calcium levels had larger hematoma volumes and higher neurological stroke scores at admission [15]. Along the same lines, Morotti et al. (2016) investigated whether a low serum calcium level is associated with an increase in the extent of bleeding in patients with intracerebral hemorrhage, measured by baseline hematoma volume and risk of hematoma expansion [16]. The researchers observed patients had a higher median baseline hematoma volume than normocalcemic patients [16]. They concluded that low calcium levels may be associated with a subtle coagulopathy predisposing to increased bleeding and might therefore be a promising therapeutic target for acute intracerebral hemorrhage treatment protocols. A recent systematic review and meta-analysis confirmed the prognostic role of serum calcium for intracerebral bleeding [17].

The clinical importance of serum calcium could also be transposed in the cardiovascular field and other postoperative patients. In postoperative orthopedic patients, Wang et al. (2021) found that hypocalcemic patients had more total blood loss in elderly patients with hip fracture, along with larger differences in Hb and Hct levels from admission to postoperative day [18]. Even after adjusting for potential confounding factors, hypocalcemia still played an independent role in blood loss and led to an almost 50 mL increase in total blood loss [18]. Presently, the study of Li et al. remains the only piece of evidence that correlates hypocalcemia with bleeding in post-major cardiac surgery patients [7]. The primary finding of our study is that lower preoperative ionized calcium levels were associated with a non-significant trend toward increased postoperative bleeding, greater use of medical hemostatic agents, and higher red blood cell transfusion requirements in the first 48 h after major cardiac surgery. While some associations reached nominal *p*-values < 0.05, none met the Bonferroni-corrected threshold of *p* < 0.00625, and therefore should be interpreted with caution.

While calcium fluctuations are common during CPB, their direct influence on bleeding outcomes remains variable [19,20,21,22]. Maintaining calcium homeostasis is critical for optimal outcomes in cardiac surgery, but the relationship between calcium levels and postoperative bleeding is complex and not fully understood. While hypocalcemia during CPB can contribute to complications, its direct effect on bleeding remains inconclusive. Therefore, individualized calcium management strategies considering both the risks of hypocalcemia and hypercalcemia are recommended to improve patient outcomes.

Postoperative ionized calcium levels, measured immediately after cardiopulmonary bypass (CPB) and during the first 24 h postoperatively, were not associated with bleeding outcomes. This suggests that transient calcium fluctuations during CPB may not independently influence early postoperative hemostasis or that any effect was too subtle to detect in our relatively small cohort.

Vitamin D plays a critical role in calcium absorption and homeostasis, which is essential for the coagulation cascade. Deficiency may lead to subtle hypocalcemia, impaired platelet function, and altered coagulation factor activity, potentially predisposing patients to increased bleeding. Similarly, lower serum vitamin D levels showed suggestive associations with increased postoperative blood loss and greater need for hemostatic intervention. These findings align with the known physiological role of vitamin D in calcium absorption and calcium’s critical role as a cofactor in coagulation. However, these trends did not reach adjusted statistical significance and should be viewed as hypothesis-generating rather than conclusive.

While some associations between calcium or vitamin D levels and bleeding outcomes were observed, these did not remain significant after Bonferroni adjustment. Therefore, these results should be regarded as hypothesis-generating rather than clinically definitive, and require confirmation in larger, adequately powered prospective studies. Our findings are consistent with prior studies highlighting the potential influence of preoperative calcium status on bleeding in other surgical contexts, including orthopedic and neurosurgical patients. However, the limited number of patients with clinically significant hypocalcemia, the retrospective design, and the single-center nature of our study limit the ability to draw firm conclusions.

Overall, these results highlight the potential role of preoperative calcium and vitamin D status as modifiable risk factors for bleeding in major cardiac surgery, but further prospective studies with larger samples are required to confirm these associations and clarify their clinical relevance.

### Limitations

This study is limited by its observational nature, which, despite multivariable adjustment for multiple potential confounders, predisposes it to residual and unobserved confounding. The observational nature also precluded establishment of causality. Smaller effects would not have been reliably detected, confirming that our study was underpowered for modest associations. Additionally, the small sample and relatively low event rates for some of the outcomes limited the reliability of our findings, as evident from the wide 95% CIs. The single-center nature also limited the findings’ generalizability.

Hypocalcemia was treated with calcium infusion, and we had just a small number of cases, so we did not analyze them separately. In addition, the relatively small sample limited the statistical power of our analyses. The post hoc power analysis indicated that with 83 patients, the study had 80% power to detect only relatively large differences (≈90 mL in drainage volume), whereas smaller but potentially clinically relevant associations may have been missed. This limitation is also reflected in the wide confidence intervals observed in some regression models. Therefore, our findings should be interpreted as exploratory and hypothesis-generating, warranting confirmation in larger prospective cohorts. Larger prospective studies addressing the above issues are warranted. The modest cohort size limited statistical power and contributed to wide confidence intervals. Post hoc analyses indicated that with 83 patients we had ~80% power to detect only relatively large effects: ~90 mL in drainage volume for continuous outcomes, and for the binary outcome of red blood cell transfusion, differences in the order of ~34% vs. ~66% (OR ~3.8) at α = 0.05, with even larger detectable effects required after Bonferroni correction (e.g., ~34% vs. ~72%; OR ~6.2). Consequently, smaller, but clinically relevant associations may have gone undetected, and our results should be interpreted as exploratory and hypothesis-generating.

Another limitation is that ionized calcium was not measured directly, but calculated from serum calcium and protein. While this surrogate method has been applied in previous clinical research, it is inherently less accurate than direct measurement and may introduce measurement error. Such misclassification would most likely bias associations toward the null, meaning that any true relationships between calcium levels and bleeding outcomes could have been underestimated.

Finally, some associations between calcium or vitamin D levels and bleeding-related outcomes did not remain statistically significant after Bonferroni adjustment. While these exploratory findings may suggest potential biological relationships, they should be regarded as hypothesis-generating rather than clinically definitive. Larger, adequately powered prospective studies are needed to confirm whether these associations reflect true causal effects.

## 5. Conclusions

In our study, lower preoperative ionized calcium levels demonstrated exploratory associations with increased bleeding-related outcomes following major cardiac surgery, including greater use of medical hemostatic agents and red blood cell transfusions. Ionized calcium levels measured immediately after cardiopulmonary bypass (CPB) did not appear to influence bleeding outcomes. Additionally, lower serum vitamin D levels showed exploratory associations with increased postoperative blood loss and a higher likelihood of requiring hemostatic support. These findings are hypothesis-generating and require confirmation in larger, adequately powered studies.

The role of calcium in coagulation homeostasis during cardiac surgery remains an important area for future research, particularly given the ionized calcium fluctuations induced by cardioplegia and CPB. Further studies with larger cohorts are needed to clarify these potential associations. Our findings suggest a possible relationship between lower preoperative ionized calcium levels, vitamin D deficiency, and increased bleeding-related outcomes, but these associations are exploratory and must be interpreted cautiously. Larger studies are warranted to establish clinical significance.

In this single-center retrospective study, lower preoperative ionized calcium levels were associated with non-significant trends toward increased postoperative bleeding, transfusion requirements, and use of medical hemostasis in patients undergoing major cardiac surgery. Postoperative ionized calcium levels measured immediately after CPB or during the first postoperative day did not influence bleeding outcomes. Lower vitamin D levels also showed suggestive associations with increased blood loss.

These findings suggest that preoperative calcium and vitamin D status may influence early postoperative bleeding, but the associations were not statistically significant after Bonferroni correction. 

## Figures and Tables

**Table 1:** Baseline characteristics of the study population.

Variable	Value (N = 83)
Age (years)	64.9 ± 8.5
Males (n, %)	49 (59%)
Weight (kg)	82 ± 5
Procedure	
Coronary procedure	26 (31.3%)
Valve procedure	42 (50.6%)
Mixed procedure (valve + coronary)	8 (9.6%)
Aortic valve and ascending aorta repair	5 (6%)
Other (ASD correction, etc.)	2 (2.41%)
Dyslipidemia	18 (21.6%)
Hypertension	23 (27.7%)
CKD	18 (21.6%)
Chronic pulmonary disease	8 (9.6%)
Peripheral artery disease	9 (10.8%)
Chronic anticoagulation	19 (22.8%)
Aortic clamp time (min)	82.96 ± 0.24
Preoperative ionized calcium (mmol/L)	1.226 ± 0.23
Preoperative vitamin D (ng/mL)	17.01 ± 0.1

ASD, atrial septal defect; CKD, chronic kidney disease.

**Table 2:** Summary of regression results. All regressions displayed were adjusted for age, body mass index, sex, type of procedure, and duration of cardiopulmonary bypass.

Outcome	Summary Statistics	Immediate Postoperative Ionized Calcium Level (Value Measured After CBP)	Average First Day After Surgery Postoperative Day 0 Ionized Calcium Level
Day 1 drainage volume (mL)	Coefficient [95% confidence interval]	−1042 [−1883–−201], *p* = 0.016	−1183 [−2089–−278], *p* = 0.011
Day 2 drainage volume (mL)		−829 [−1465–−193], *p* = 0.011	−579 [−1291–133], *p* = 0.110
Requirement for surgical hemostasis	Odds ratio [95% confidence interval]	0.25 [0.00–2.76 × 10^8^], *p* = 0.896	0.00 [0.00–923.53], *p* = 0.088
Requirement for red blood cell transfusion		0.00 [0.00–0.07], *p* = 0.015	0.01 [0.00–200.47], *p* = 0.353
Requirement for fresh frozen plasma transfusion		0.00 [0.00–33.69], *p* = −0.201	0.00 [0.00–5.06], *p* = 0.086
Requirement for platelet transfusion		0.00 [0.00–30432.16], *p* = 0.340	0.00 [0.00–17680.86], *p* = 0.212
Requirement for medical hemostasis		0.00 [0.00–16.48], *p* = 0.145	0.00 [0.00–0.07], *p* = 0.024
Extracardiac haemoragic complications		0.00 [0.00–3.92 × 10^7^], *p* = 0.360	0.00 [0.00–9.72 × 10^6^], *p* = 0.212

## Data Availability

The data supporting the findings of this study are available from the corresponding author upon reasonable request. Due to ethical and privacy restrictions related to patient confidentiality, the datasets generated and/or analyzed during the current study are not publicly available. Anonymized data may be provided to qualified researchers subject to institutional approval and compliance with applicable data protection regulations.

## Supplementary Materials

The following supporting information can be downloaded at: https://www.mdpi.com/article/10.3390/life15091460/s1, Table S1. Postoperative bleeding outcomes stratified by vitamin D status.
