# Supplementary material for: The Relationship Between Preoperative Serum Ionized Calcium, Vitamin D, and Postoperative Bleeding After Major Cardiac Surgery
Source: Life (Basel). 2025 Sep 17;15(9):1460. doi: 10.3390/life15091460 (PMC12471322; doi:10.3390/life15091460)
Supplement: Supplementary file 1 [file life-15-01460-s001.zip › life-3846857-Supplementary materials.pdf]

Table S1. Postoperative bleeding outcomes stratified by vitamin D status

Vitamin D status	N	Median Day 1 Drainage (mL, IQR)	Median Day 2 Drainage (mL, IQR)	RBC Transfusion n (%)	Medical Hemostasis n (%)
Deficient (<20 ng/mL)	45	460 (380–520)	310 (250–370)	18 (40%)	9 (20%)
Insufficient (20–30 ng/mL)	30	400 (350–480)	270 (220–330)	9 (30%)	4 (13%)
Normal (30–50 ng/mL)	8	380 (350–420)	240 (200–280)	1 (12%)	1 (12%)

Notes:

- RBC = red blood cell transfusion; IQR = interquartile range.
- Values are presented as median (IQR) for continuous outcomes and n (%) for categorical outcomes.
- This stratified analysis is exploratory due to small sample sizes, particularly in the normal vitamin D group, and should be interpreted as hypothesis-generating.

"Vitamin D levels were categorized as deficient (<20 ng/mL), insufficient (20–30 ng/mL), or normal (30–50 ng/mL). Bleeding outcomes were compared across these categories using descriptive statistics, and results are presented in Supplementary Table S1."
